# Fluorescent Materials With Aggregation-Induced Emission Characteristics for Array-Based Sensing Assay

**DOI:** 10.3389/fchem.2020.00288

**Published:** 2020-04-24

**Authors:** Engui Zhao, Puxiang Lai, Yongjun Xu, Gang Zhang, Sijie Chen

**Affiliations:** ^1^School of Chemical Engineering and Energy Technology and Engineering Research Center of None-food Biomass Efficient Pyrolysis and Utilization Technology of Guangdong Higher Education Institutes, Dongguan University of Technology, Dongguan, China; ^2^Department of Biomedical Engineering, The Hong Kong Polytechnic University, Hong Kong, China; ^3^Ming Wai Lau Centre for Reparative Medicine, Karolinska Institutet, Hong Kong, China

**Keywords:** aggregation-induced emission, array-based sensing, biological sensing, bacteria identification, sensor array

## Abstract

Array-based sensing is a powerful tool for identifying analytes in complex environments with unknown interferences. In array-based sensing, the sensors, which transduce binding details to signal outputs, are of crucial importance for identifying analytes. Aggregation-induced emission luminogens (AIEgens) enjoy the advantages of easy synthesis and high sensitivity, which enable them to facilely form a sensor pool through structural modifications and sensitively reflect the subtle changes associated with binding events. All these features make AIEgens excellent candidates for array-based sensing, and attempts have been made by several research groups to explore their potentials in array-based sensing. In this review, we introduce the recent progresses of employing AIEgens as sensors in sensing assays and in building up sensor arrays for identification of varied biological analytes, including biomolecules and bacteria. Examples are selected to illustrate the working mechanism, probe design and selection, capability of the sensor array, and implications of these sensing methods.

## Introduction

In real world, identification of unknown analyte is a very challenging task, which requires precise recognition of targeted analyte in complicated context with unknown interferences. The situation is even worse when the interferences share some similarities with the analyte. Traditional detection methods mostly respond to one specific target; thus, multiple methods are needed for identifying an unknown sample until one testing method gives positive results. The combination of statistics and sensing has been proven to be a very successful attempt for identifying analytes in complicated environment, a good example of which is array-based sensing (Miranda et al., 2007, 2010b; Bajaj et al., 2010; Escobedo, 2013). In array-based sensing, there are three processes: recognition, transduction, and data analysis (Le et al., 2014). Sensors bind to targets through selective or specific binding, which produces a detectable signal. The signal can then be detected and processed with varied statistical methods and produce a two-dimensional (2D) or three-dimensional (3D) dataset that separates analytes to different regions with clear boundary. As such, unknown analytes can be quickly identified by similar binding, transduction, and data analysis processes. In array-based sensing, several statistical methods are usually employed to process the data produced, such as hierarchical cluster analysis (HCA), principal component analysis (PCA), and linear discriminant analysis (LDA). In HCA, samples are grouped according to their similarity, which is usually computed through a user-defined similarity matrix. Samples with high similarities merge into groups, whereas those with low similarities are separated into different groups. Principal component analyses also calculate the intercorrelations between different samples. It then compresses the dimensional vectors to extract principle components that retain most of the information. Linear discriminant analysis works on the basis of Fisher criterion, which finds the vector that best distinguishes among classes.

**Figure 1:**
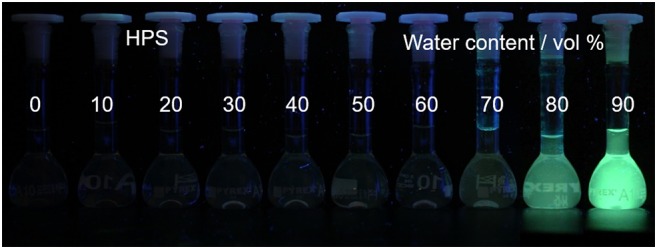
Fluorescence photographs of HPS in THF/water mixtures with different water contents. Adapted from Mei et al. (2015) with permission. Copyright 2015 American Chemical Society.

**Figure 2:**
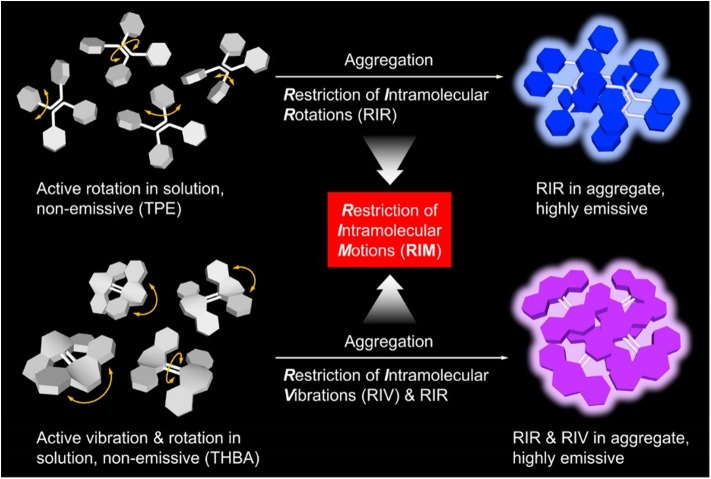
Illustration on the RIR process of TPE and the RIR and RIV processes of THBA. Reproduced from Zhu et al. (2018), https://pubs.acs.org/doi/10.1021/acsabm.8b00600, with permission. Copyright 2018 American Chemical Society.

In these array-based sensing, fluorescent materials are widely used as sensors to uncover the binding processes, due to their advantages of high sensitivity and selectivity, simple operation, and feasibility to sense varied analytes through molecular engineering. However, conventional fluorescent materials are usually featured with large conjugated coplanar structures, which can easily undergo strong π-π stacking interactions and non-radiatively decay the excited-state energy. For this reason, conventional fluorescent materials are usually good fluorescence emitters in dilute solution, but become weakly or non-emissive at high concentrations or in the particle or solid state. The phenomenon is generally referred to as aggregation-caused quenching effect. Aggregation-caused quenching effect is not the favorite for many real-world applications, as most practical applications are in the solid state or as aggregates when no solvent is involved. In 2001, Tang et al. reported a new species of luminescent materials that demonstrated a completely different fluorescence behavior: a species of propeller-shaped luminogens, such as tetraphenylethylene (TPE) and hexaphenylsilole (HPS), emitted faintly in dilute solutions, but fluoresced intensely in the aggregated state (Luo et al., 2001; Hong et al., 2009, 2011; Mei et al., 2014, 2015). Taking HPS as an example, its tetrahydrofuran (THF) solution is non-emissive (Figure 1) (Mei et al., 2014). When the bad solvent, water, is introduced to the solution, the solvation power of solvent mixture decreases gradually. At a water fraction of 70%, HPS starts to form aggregates, and its fluorescence can be discerned. Further increasing water fraction to 90% leads to bright fluorescence. They termed the phenomenon as aggregation-induced emission (AIE). Through systematic study, its mechanistic picture was unveiled (Figure 2) (Zhu et al., 2018). The phenyl rings of AIE molecules are usually twisted from the fluorogen and can undergo dynamic rotations in solutions, which non-radiatively decay the excited-state energy and lead to weak or no emission in the solutions. In the aggregated state, such molecular motions are restricted because of the spatial constraints from adjacent molecules. Besides, the twisted molecular conformation prevents strong π-π stacking interactions from taking places, and thus, the excited-state electrons can decay through radiative pathway, resulting in fluorescence. Thus, restriction of intramolecular rotation (RIR) accounts for the AIE feature of these materials. Besides, some AIE molecules can vibrate in solutions, such as tetrahydro-5,5′-bidibenzo[a,d][7] annulenylidene (THBA). The vibrational motions are also restricted in the aggregated state, resulting in the enhanced fluorescence. The process is termed as restriction of intramolecular vibration (RIV). Collectively, RIR and RIV comprise restriction of intramolecular motions (RIMs), which mainly accounts for the AIE effect. Materials with AIE characteristics draw the attention of researchers from all around world and have become a hot research topic. With the continuous endeavor, lots of their applications in chemical sensing, bioimaging, and photodynamic therapy have been made (Dai et al., 2000; Ding et al., 2013; Li et al., 2013; Kwok et al., 2014; Lou et al., 2016; Zhan et al., 2016; Qian and Tang, 2017). Recently, their applications in array-based sensing have also been explored. In this review, we will first introduce a few AIE-based sensors and their applications in sensing assays, followed by introducing the recent progresses of the applications of AIE sensors in array-based sensing.

## AIE-Based Sensing assay

Aggregation-induced emission materials are excellent candidates for constructing fluorescence turn-on sensors. Various AIE-based biosensors have been developed in recent years. Taking advantage of their high sensitivities, a variety of biological assay systems have been successfully developed, which includes the enzyme assays such as the sialidase, RNA microchip, and bacteria assay for antibiotics screening.

### Sialidase Detection and Inhibitor Screening

Sialidase, also known as neuraminidase, widely exists in virus, bacteria, and mammalian cells. Sialidase can cleave a sialic acid residue from sialoglycoconjugates, such as glycoproteins and glycolipids, and plays important roles in many physiological and pathological processes (Minami et al., 2014; Rivas et al., 2019), such as spreading of virions during virus evasion and facilitating the penetration of toxin into intestinal mucosal cells. Besides, sialidase is also a characteristic enzyme that is produced during bacterial vaginal infections and is treated as an important marker of bacterial vaginosis (BV) for clinical diagnosis. Compared with traditional bacteria culture-based method for diagnosing BV, detection of sialidase is a fast, simple, and inexpensive method. By targeting sialidase, some small molecules can inhibit the enzymatic activity of sialidase and function as drug for influenza infection, such as oseltamivir and zanamivir. In this sense, detecting sialidase, and screening of sialidase inhibitors may greatly contribute to the diagnosis of BV and development of drugs for sialidase-related diseases, respectively (Kurebayashi et al., 2014).

**Figure 3:**
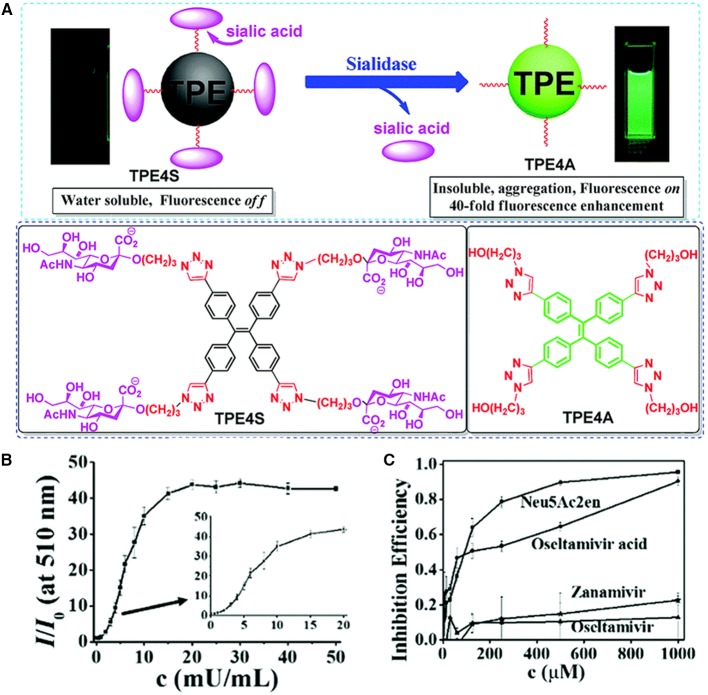
**(A)** The working principle of employing TPE4S for detection of sialidase and molecular structures of TPE4S and TPE4A. **(B)** Fluorescence responses of TPE4S (20 μM) to various concentrations of sialidase. TPE4S was incubated with sialidase in PBS (pH 7.1, 6.7 mM) for 1 h before measuring the fluorescence intensity. **(C)** The inhibition effect of some potential inhibitors evaluated with TPE4S using a 96-well plate assay. *I* and *I*_C_ represent the fluorescence intensities with and without potential inhibitors, respectively, and *I*_0_ is the fluorescence intensity of 20 μM TPE4S itself. λ_em_ = 510 nm. Reproduced from Liu et al. (2018) with permission. Copyright 2018 Royal Society of Chemistry.

Liu et al. (2018) reported a fluorescence-based method for the detection of sialidase activity by employing an AIE-active sensor (Figure 3). Taking advantage of the click reaction between azide group and triple bond, they conjugate four sialic acids to TPE to prepare TPE4S. The four sialic functionalities of TPE4S endow it with good water solubility. Thus, TPE4S does not fluoresce in aqueous solution, because the vigorous rotations of its phenyl ring decay the excited-state energy non-radiatively. Sialidase can cleave the sialic acid functionalities from TPE4S, resulting in the formation of TPE4A. Without sialic acid functionalities, TPE4A is a hydrophobic molecule and trends to form aggregates in aqueous solution. This blocks the non-radiative decay pathway of the excited state electron and allows it to decay radiatively, resulting in the fluorescence of TPE4A. In this way, the presence of sialidase can be visualized in a fluorescence turn-on manner. As shown in Figure 3B, upon incubating with sialidase at 37°C for 1 h, the fluorescence emissions of TPE4S solution monotonically increase as the concentration of sialidase is increased from 0 to 50 mU mL^−1^. Extending the incubation time to 2 h allows the detection of sialidase in the concentration range of 0 to 6 mU mL^−1^. The detection range can be further lowered by increasing the incubation time. At an incubation time of 24 h, the detection limit is 0.1 mU mL^−1^. The authors further employ the method for high-throughput screening of sialidase inhibitors. Some well-known inhibitors, such as *N*-acetyl-2,3-dehydro-2-deoxyneuraminic acid (Neu5Ac2en), zanamivir, and oseltamivir acid (the active ingredient of the precursor drug oseltamivir), are examined of their inhibition effect. As plotted in Figure 3C, Neu5Ac2en and oseltamivir acid demonstrate high inhibition efficiency, with low half maximal inhibitory concentrations (IC_50_) of 73 and 98 μM, respectively, whereas zanamivir and oseltamivir exhibit low inhibitory effect. The results are consistent with previous report, which substantiate the reliability of this method in screening sialidase inhibitors. The authors further employ the method for high-throughput fluorescence-guided diagnosis of BV, and similar results are obtained as BVBlue, which is the standard method for clinical BV diagnosis.

### MicroRNA Detection

MicroRNAs (miRNA) are short RNA molecules that can bind to messenger RNA (mRNA), resulting in the translational repression of the corresponding mRNA. It plays important roles in the regulation of cell functions, such as development, differentiation, growth, and metabolism. The mRNA level should be precisely controlled, and both down-regulation and up-regulation of the corresponding gene will lead to serious consequences, such as cancer, cardiovascular disease, cardiac failure, inflammatory disease, neurodevelopmental disease, autoimmune disease, liver disease, and skin disease (Ardekani and Naeini, 2010). On the other hand, miRNA can serve as effective cure for cancer, with several miRNA-targeted therapeutics reaching clinical development (Rupaimoole and Slack, 2017). Detection and quantification of miRNA are thus of crucial importance in disease diagnosis and treatment.

**Figure 4:**
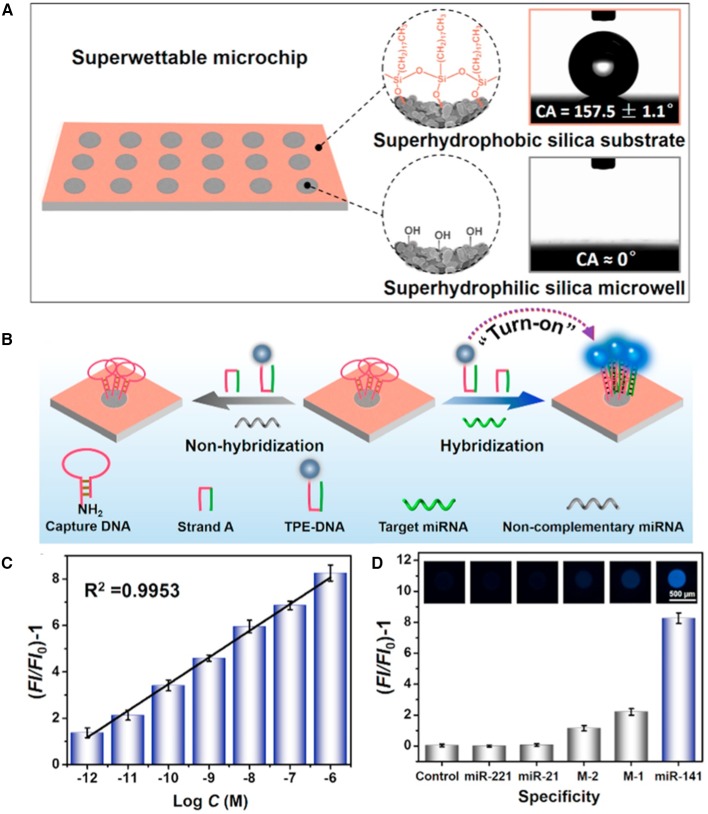
**(A)** Illustrations of surface functionalities of the superwettable microchip. Octadecyltrichlorosilane-modification endows the substrate with superhydrophobicity with a water contact angle (CA) of around 157.5°, while irradiation destroys the OTS modification and makes microwell superhydrophilic with a water CA approaching 0°. **(B)** Design of miRNA detection by employing AIEgen-based superwettable microchips. **(C)** Fluorescence responses of AIEgen-based microchips toward different concentrations of miR-141. **(D)** Assessment on the specificity of the AIEgen-based microchips for the detection of miR-141. Reproduced from Chen et al. (2018) with permission. Copyright 2018 Elsevier.

Xu et al. reported a superwettable microchip based on AIEgen for biosensing and demonstrated its feasibility for quantifying miRNA (Chen et al., 2018). In their approach, high sensitivity was achieved by taking advantages of the synergetic effect of evaporation-induced enrichment and AIE. Through confining the targeted miRNA and TPE-DNA onto the microwells, followed by evaporation of water, miRNA and TPE-DNA were enriched in a small spot. Thanks to the AIE effect of TPE-DNA, the fluorescence of TPE-DNA was greatly enhanced in this process, and high sensitivity can be achieved. They first decorate a candle soot coating with tetraethyl orthosilicate through chemical vapor evaporation, which is then modified by octadecyltrichlorosilane (OTS) to prepare a superhydrophobic surface with a water contact angle (CA) of 157.5° ± 1.1°. The superhydrophobic surface is then irradiated under UV light through a photomask to decompose the OTS in exposed regions, which produces superhydrophilic silica microwells with a water CA of around 0°. In this way, a superhydrophilic microwell array on superhydrophobic surface is prepared (Figure 4A). As aqueous droplets containing analytes are added to the microarray, they will be confined to the microwell, due to the great hydrophilicity difference of the microwells and the substrate. Upon evaporation of the water, samples will be enriched in the bottoms of the mircroarrays, which amplifies the detection signal. Thus, microwells with small diameter will enrich the analytes to a larger extent than large microwells do. Once the microwells are modified with capture DNA, a selective miRNA detection microarray is produced. In the presence of targeted miRNA, which is miR-141 in their experiment, it will form a four-way junction structure with capture DNA and TPE-DNA and capture the miR-141 and TPE-DNA to the microwell. After washing and evaporation of water, the fluorescent sensor, TPE-DNA, will be enriched and lit up due to the RIM in the solid/concentrated state. By employing this strategy, low miR-141 detection limit of 1 pM is achieved, demonstrating the sensitive detection for target analyte by the AIE-based superwettable microchip (Figure 4B). Due to the high specificity of the recognition, other interfering miRNAs, including those with one (M-1) or two (M-2) mismatches, do not show positive results (Figure 4C).

### Antibiotics Screening and Bacteria Susceptibility Evaluation

Antibiotics are regarded as one of the greatest discoveries in twentieth century. By fighting against bacteria, they are important antibacterial agents for clinical treatment of infections (Ling et al., 2015). However, as the antibiotic-resistant issue arises, antibiotics are becoming increasingly impotent in face of bacterial infections (Koehn and Carter, 2005; Pitout and Laupland, 2008). To tackle this issue, increasing efforts are spent to develop new antibiotics, which is usually an expensive and long-term process with current culture-based method. Besides, sensible selection of antibiotics is another feasible way for preventing the exacerbation of the antibiotic-resistant issue.

**Figure 5:**
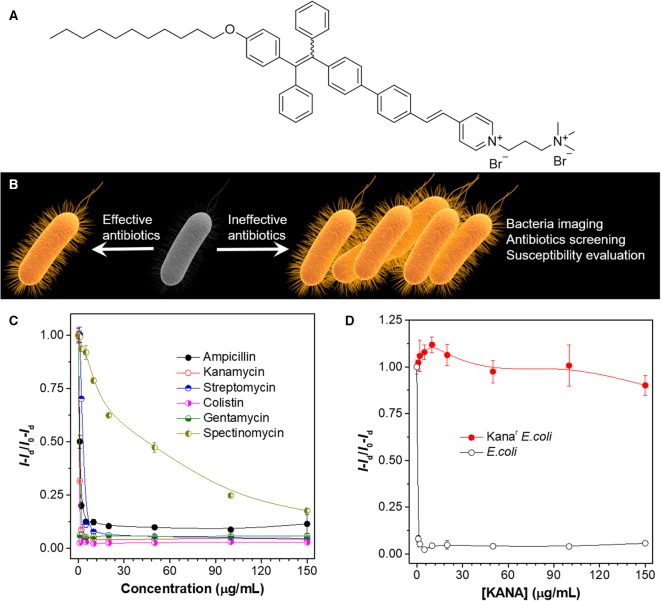
**(A)** Molecular structure of the AIEgen employed. **(B)** Illustration of the high-throughput antibiotics screening strategy. **(C)** Evaluation of the inhibition effect of different antibiotics on *S. epidermidis*. *Staphylococcus epidermidis* is incubated with different concentrations of antibiotics for 4 h, followed by quantification with the AIEgen. **(D)** Evaluation of the susceptibility of *E. coli* and Kana^r^ *E. coli* toward KANA. λ_ex_: 430 nm. Adapted from Zhao et al. (2015) with permission. Copyright 2015 John Wiley and Sons.

Tang et al. reported a fluorescence-based method for fast screening of antibiotics and evaluation of antibiotic resistance Zhao et al. (2015). The molecular structure of the AIEgen employed is shown in Figure 5A. The AIEgen is non-emissive in the solution; binding to bacteria can turn on its fluorescence. By taking advantage of the AIE characteristics, a sensing method is developed as follows: bacteria are incubated with different antibiotics for a short period of time (4 h in this study). Effective antibiotics can inhibit the growth of bacteria, whereas in ineffective antibiotics, bacteria growth is not influenced. At the end of bacteria culture, AIEgen is aliquoted to the solution. Bacteria incubated with effective antibiotics exhibit low fluorescence, whereas bacteria co-cultured with ineffective bacteria display strong emission (Figure 5B). Based on the differences in fluorescence intensity, the effectiveness of the antibiotics can be evaluated, and critical parameters for antibiotics evaluation can be obtained, such as minimal inhibitory concentration (MIC) and half maximal inhibitory concentration (IC_50_). As shown in Figure 5C, after incubating *Staphylococcus epidermidis* with varied bacteria at different concentrations and subsequently addition of the AIEgen, plots of relative fluorescence intensity vs antibiotic concentrations can be obtained, from which MIC and IC_50_ can be facilely determined within 5 h. The method can also be applied to evaluation of bacteria susceptibility. To demonstrate this, two bacteria strains, *Escherichia coli* and Kana^r^ *E. coli* (kanamycin-resistant *E. coli*), are employed for the experiments. Upon incubation with kanamycin, *E. coli* demonstrates faint emission even at low concentrations, whereas Kana^r^ *E. coli* fluoresces intensely at all the tested concentrations. The fluorescence characteristics are consistent with the susceptibility of the employed bacteria. The results clearly prove the feasibility of employing the AIEgen for evaluation of bacteria susceptibility, which may contribute to personalized therapy of infection by identifying the susceptibility of the pathogens and assisting in the wise selection of antibiotics.

## AIE Array-Based Sensing Assay

AIEgens, as they have been previously defined with distinct properties can be easily synthesized through structural modifications. These AIEgens show different responses and varied binding abilities to different targets. These features make AIEgens excellent candidates for creating sensor pools that are used for array-based sensing. Several research groups start to explore their application potentials in array-based sensing.

### Sensing of Biomolecules

Biomolecules are basic constituents of all the organisms, which lay the foundation for their biochemical functions. The level of biomolecules usually lies in a reasonable range, and their irregular concentrations are mostly associated with abnormal metabolism. Detection and quantification of biomolecules are of crucial importance for human beings, as they are direct reflections of human health state. Biomolecules exist in a large variety, and some of them possess very similar structures, which make their identification and quantification very challenging. With sensor array using AIE materials as fluorescent indicators, successful attempts have been made to identify these biomolecules.

#### Protein Sensing

Protein is one of the most important biomacromolecules, which accounts for varied biological functions, including basic building unit for biological architecture, carrier for transporting biomolecules, enzyme for metabolism, immunity for defensing foreign molecules/organisms, and regulator for physiological activity (Miranda et al., 2007, 2010a). Thus, detecting the existence and quantity of protein is of paramount importance. Proteins exist in large variety and possess structural complexity, which add to the difficulties in their identification. Currently, most protein detections are based on the “lock and key” strategy, which utilizes the specific antigen–antibody or aptamer–antibody interactions. Such strategies are effective in protein sensing, but require tremendous research effort to build up antibody/aptamer pool for different proteins. The antibody or aptamer may also become invalid in time of protein mutations. Facile methods for quick and accurate protein identification are highly desirable.

**Figure 6:**
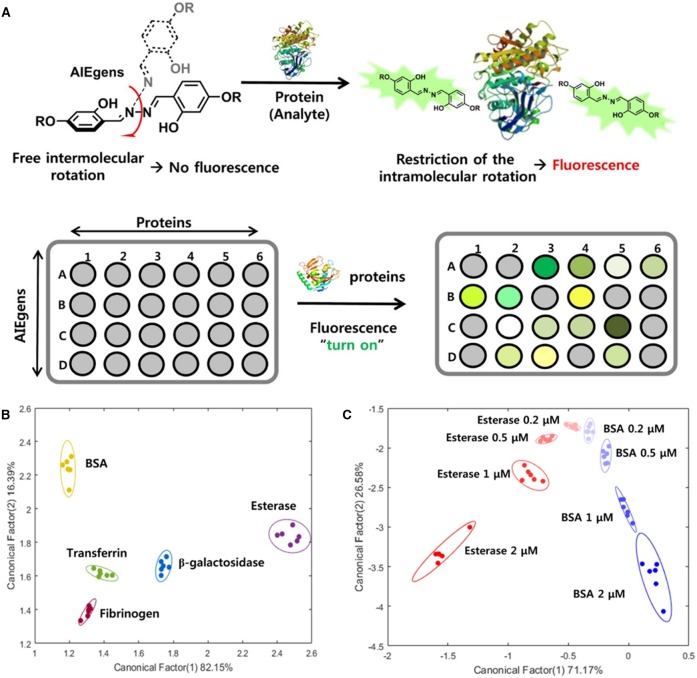
**(A)** Mechanism illustrations of the fluorescence turn on detection of proteins using an array with AIEgens as reporter. **(B)** Linear discriminant analysis of the fluorescence responses of synthesized AIEgens in the presence of the five proteins using two-dimensional (2D) plots with 95% ellipse confidence. **(C)** Linear discriminant analysis of the fluorescence responses of AIEgens in the presence of BSA and esterase at 200 nM, 500 nM, 1 μM, and 2 μM, using 2D plots with 95% ellipse confidence. Reproduced from Choi et al. (2018), https://pubs.acs.org/doi/10.1021/acsomega.8b01269, with permission. Copyright 2018 American Chemical Society.

Choi et al. (2018) developed a fluorescent array for protein sensing by using AIEgens as the fluorescent reporters. In their test, an AIEgen, di-2,2′dihydroxylbenzoylhydrozone, is selected as the fluorophore and is functionalized with different groups, such as acetate (AIE-1), trimethylamine (AIE-2), dimethylamine (AIE-3), and sulfonate (AIE-4), to create positively charged, neutral, and negatively charged molecules. Five proteins with different molecular weights (~66–540 kDa) and isoelectric points (pI: 4.6–6.3) are selected as targeted proteins, which are bovine serum albumin (BSA), esterase, transferrin, fibrinogen, and β-galactosidase. These AIEgens can randomly bind to proteins with varied affinities, arising from the different electrostatic and hydrophobic interactions involved (Figure 6A). Strong interactions ensure tighter binding and restrict the motions of AIEgens to a large extent, and thus the AIEgens exhibit bright emission, whereas weak interactions mean loose binding and allow for more free motions of the AIEgens, which endow the AIEgens with weak emissions. As a result, when mixing the four AIEgens with different proteins, distinct fluorescence patterns are formed for each protein. Based on the distinct fluorescence responses, LDA can be performed to discriminate and identify the protein patterns. By maximizing the separation between classes and minimizing the variance within classes, LDA contributes to differentiation of targeted proteins. As shown in the 2D LDA score plots of the first two factors (Figure 6B) with 95% ellipse confidence, the five proteins are well-separated from each other. As in real-world identification of proteins, the amount of protein may vary. Thus, the authors also explored the feasibility of sensing proteins with different concentrations. In the 2D plot of the first two canonical factors with 95% ellipse confidence (Figure 6C), esterase and BSA at different concentrations are distinctly separated from each other. The results demonstrate the facile applications of employing these AIEgens for array-based sensing of proteins.

#### Differentiation of Amino Acid

Amino acids are the building blocks for protein. Although hundred species of amino acids exist in nature, only 20 of them are found in proteins, featuring an α carbon bonded to a hydrogen atom, a carboxyl group, an amino group, and an R group. Although the R groups vary from polar, non-polar, to charged, precise identification of them remains challenging, partially due to their large quantity and structural similarity.

**Figure 7:**
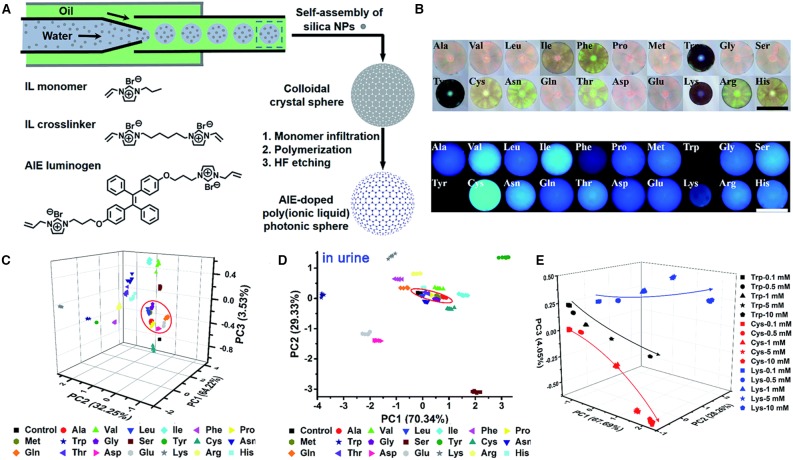
**(A)** Procedures for preparing AIE-doped poly(ionic liquid) photonic sphere and the molecular structures of employed ionic liquid monomer, crosslinker, and AIEgen. **(B)** The optical and fluorescence images of the AIE-doped poly(ionic liquid) photonic spheres (OH^−^ form) to 20 natural amino acids (10 mM). **(C,D)** Three-dimensional PCA plot of AIE-doped poly(ionic liquid) photonic spheres of the OH^−^ form for the discrimination of 20 natural amino acids at 10 mM in **(C)** water and **(D)** urine. **(E)** Three-dimensional PCA plot of the semiquantitative assay of Trp, Cys, and Lys at five different concentrations by the AIE-doped PIL photonic spheres of the OH^−^ form. Reproduced from Zhang et al. (2017) with permission. Published by The Royal Society of Chemistry. Copyright 2017 Royal Society of Chemistry.

Zhang et al. (2017) developed a fluorescent array with multidimensional signal channels for the differential sensing of amino acids by employing AIEgen-doped poly(ionic liquid) (PIL) photonic spheres. The synthetic procedures are shown in Figure 7A. Monodisperse silica nanoparticles with a diameter of 170 nm are first prepared by droplet-based microfluidics, which then self-assemble into colloidal crystal sphere. The colloidal crystal sphere is then infiltrated with solutions of IL monomer, AIEgen, crosslinker, and photoinitiator and serves as the template for the photoinitiated polymerization. Then, the colloidal crystal sphere is treated with hydrofluoric acid to remove the SiO_2_, producing the AIE-doped inverse opal PIL spheres. The PIL spheres with macropore structure exhibit photonic properties, whereas the TPE units endow it with strong fluorescence. Both the photonic properties and TPE fluorescence are sensitive to the molecular interactions with analytes, such as Van der Waals forces, electrostatic forces, hydrogen bonding, hydrophobic interactions, and π-π interactions, and respond correspondingly to different analytes. By collecting the information of the photonic properties and TPE fluorescence, enhanced discrimination can be achieved with miniaturized array. In their approach, one TPE-doped PIL photonic sphere alone can generate enough sensing information for precisely discriminating complex systems, achieving “lab-on-a-sphere.” As shown in Figure 7B, 20 natural amino acids are applied to the PIL photonic spheres in OH^−^ form, which demonstrate distinctly different responses in both photonic and fluorescence properties. This can be attributed to the different extents of molecular interactions involved, which lead to the shrinking or swelling of the PIL photonic spheres and produce diverse wavelength shifts of Bragg diffraction and fluorescence intensity changes of TPE units. By investigating on the wavelength shift of Bragg diffraction and the fluorescence changes at 515 and 554 nm, PCA is employed to evaluate and classify these amino acids (Figure 7C). The resultant 3D PCA score plot intuitively exhibits a clear clustering of all 20 different amino acids, and the leave-one-out validation routine displays 100% accuracy for the classification of all of the 147 samples, which substantiate the excellent discriminatory power. Besides, high differential sensing of amino acids can also be achieved in complicated environment, and clear clustering is also achieved in human urine (Figure 7D). Furthermore, semiquantitative sensing of amino acids can also be achieved (Figure 7E). tryptophan, cysteine, and lysine are clearly separated at different gradient concentrations (0.1, 0.5, 1, 5, and 10 mM), whose locations distribute along the increasing concentrations.

By employing similar strategy, 9 important phosphate derivatives and 10 metal ions are also differentiated with high accuracy, which proves the strong power of this sensing method. More intriguingly, by changing the counter ion of the PIL photonic sphere, various ILs with tunable physicochemical properties can be accessed conveniently, which enables the facile preparation of a sensing platform for high-throughput sensing of varied analytes.

### Bacteria Identification

Bacteria are ubiquitous microorganisms that exist in large quantity and diversity. Although bacteria share simple structures, their activities vary to a large extent. Some bacteria are beneficial to humans, whereas others are harmful or even vital. Identification of bacteria is thus very important for their clinical treatment. Because bacteria are too small to be visualized or differentiated by naked eyes and optical microscope, they are mostly identified by the morphological appearance of their bacterial colonies, which is tedious and labor extensive and should be performed in a biosafety cabinet to avoid contamination. However, the suitable growth conditions differ among bacteria, and some bacteria are very difficult to grow; thus, this method only works well on common bacteria with well-known growth conditions and becomes impotent for bacteria that are difficult to culture. A number of biochemical kits are also available for bacteria identification, such as carbohydrate test, enzyme test, immunological test, protein and nucleic acid sequences, and typing method, which are too expensive and time-consuming for routine bacteria identification (Noble and Weisberg, 2005; Ahmed et al., 2014; Law et al., 2014; Deshmukh et al., 2016). As mentioned previously, array-based sensing is powerful in identifying subjects with subtle differences in a complex environment, so research effort has been devoted to the development of sensor arrays for identification of bacteria. Aggregation-induced emission materials with low background emission, high sensitivity to environmental changes, and easy synthesis are ideal candidates for array-based sensing, and some pioneer works have been done in this area (Liu et al., 2017).

**Figure 8:**
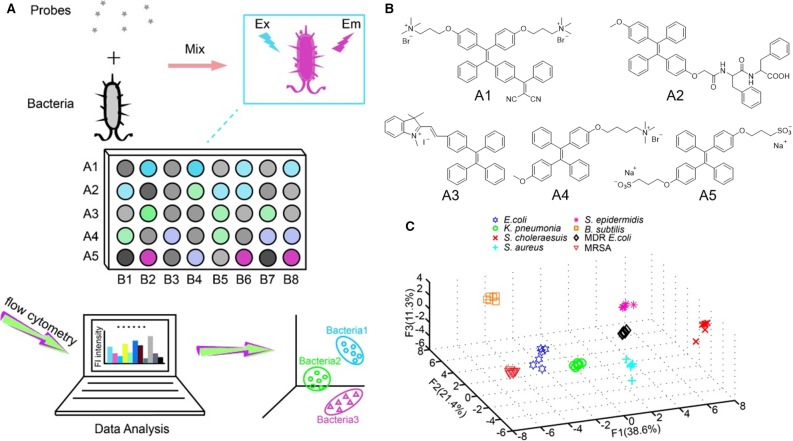
**(A)** The design principle for bacteria identification using a sensor array. A1–A5 denote five different probes, and B1–B8 represent eight bacteria species. **(B)** Molecular structures of the AIEgens employed. **(C)** Principal component analysis plot of the fluorescence responses of five AIEgens toward eight kinds of bacteria. F1, F2, and F3 are the top three rates of contribution. Reproduced from Chen et al. (2014) with permission. Copyright 2014 John Wiley and Sons.

Wang, Zhang and Jiang et al. reported an AIEgen-based fluorescent array for identification of bacteria. In their detection method, a set of fluorescent probes are incubated with different bacteria (Figure 8A) (Chen et al., 2014). Different bacteria interact differently with the fluorescent probes and produce varied fluorescence responses. By employing flow cytometry, such fluorescence responses can be collected and summarized, from which PCA can be applied to produce 2D or 3D plots. In these plots, different bacteria clusters are well-separated with clear boundary, which enables the identification of unknown samples. In array-based sensing, it is of paramount importance that the sensors respond differently to varied subjects; thus, sensors with different binding affinity and responses to the subjects are the key factors determining the accuracy of identification. Bearing this in mind, five AIEgens with different charges and functionalities are utilized for building up the fluorescent array. As shown in Figure 8B, probe A1 bears two positive charges, whereas A3 and A4 carry one positive charge each. Probe A2 and A5 are one and two negatively charged molecules, respectively. Then, the five probes are employed to stain eight different bacteria, and their fluorescence responses are collected through flow cytometry. Quadratic discriminant analysis is used to process these fluorescence responses, where the Mahalanobis distances of the new case to the respective centroids of eight groups are calculated (Figure 8C). After evaluating the fluorescent array through a cross-validation procedure, a high prediction probability of 93.75% is achieved, which demonstrates the high accuracy of this detection method. The fluorescent array can be further simplified by decreasing the number of fluorescent probes employed. By using A1 and A3 alone to distinguish the same eight kinds of bacteria through PCA, high accuracy is still maintained, which demonstrates the high identification power of this fluorescent array.

**Figure 9:**
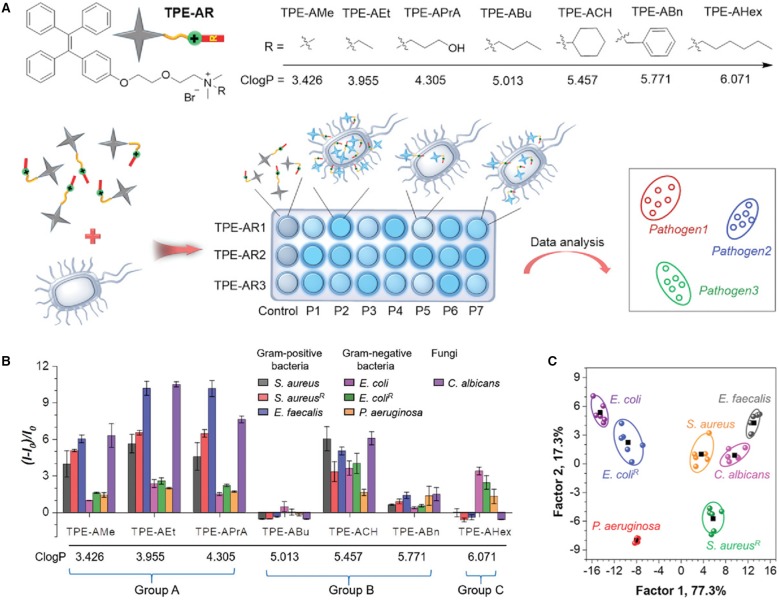
**(A)** Molecular structures of TPE-ARs and schematic illustration of pathogen identification with fluorescence sensor array. P1–P7 are seven kinds of pathogens. **(B)** Fluorescence responses of seven TPE-ARs (20 × 10^−6^ M) toward different microbes. λ_em_: 470 nm. *I*_0_ and *I* are the fluorescence intensity of TPE-ARs in the absence and presence of microbes. **(C)** Canonical score plot for the fluorescence response patterns determined by LDA. Reproduced from Zhou et al. (2019) with permission. Copyright 2018 John Wiley and Sons.

Tang and Wang et al. reported a facile sensor array-based method for identification of pathogens (Zhou et al., 2019). In their work, a series of TPE derivatives, TPE-ARs, are designed and synthesized as the fluorescent sensors (Figure 9A). These TPE-ARs are engineered to bear a tertiary amine and different hydrophobic functionalities, which fine tune their hydrophobic and electrostatic interactions with the pathogens. The hydrophobicity of these TPE-ARs are evaluated by calculated log*P* (*C*log*P*) values, which lies between 3.426 and 6.071 (log*P* is *n*-octanol/water partition coefficient, estimated using ChemBioDraw 14.0). To demonstrate the feasibility of this method, seven microorganisms were employed for the identification, which are *Staphylococcus aureus*, penicillin-resistant *S. aureus* (abbreviated as *S. aureus*^R^) and *Enterococcus faecalis, E. coli*, ampicillin-resistant *E. coli* (abbreviated as *E. coli*^R^), *Pseudomonas aeruginosa* and *Candida albicans*, which include both Gram-positive bacteria (the first three bacteria), Gram-negative bacteria (the following three bacteria) and fungus (*C. albicans*). After incubating the seven pathogens with TPE-ARs, diverse fluorescence responses are achieved (Figure 9B). Based on the differences in fluorescence responses, the TPE-ARs are classified into three groups. In Group A with 3<*C*log*P*<5, the relative fluorescence of TPE-ARs is larger for Gram-positive bacteria and fungus than Gram-negative bacteria. In Group B with *C*log*P* lying between 5 and 6, similar fluorescence responses are achieved for all the tested pathogens. In Group C with *C*log*P* larger than 6, the TPE-ARs show larger fluorescence enhancement upon incubation with Gram-negative bacteria than that with Gram-positive bacteria. These differences in fluorescence responses are reflective of the structural variations of these pathogens. To balance the detection accuracy and simplicity of sensor array, one TPE-AR in each group is selected for building up a sensor array and the sensor array of TPE-APrA, TPE-ACH, and TPEAHex is selected as an example. Through LDA, a 2D canonical score plot can be facilely generated, in which the seven pathogens cluster with well-separated from each other (Figure 9C). The high effectiveness of this detection method is proved by “leave-one-out” cross-validation in LDA with 100% accuracy of discrimination.

**Figure 10:**
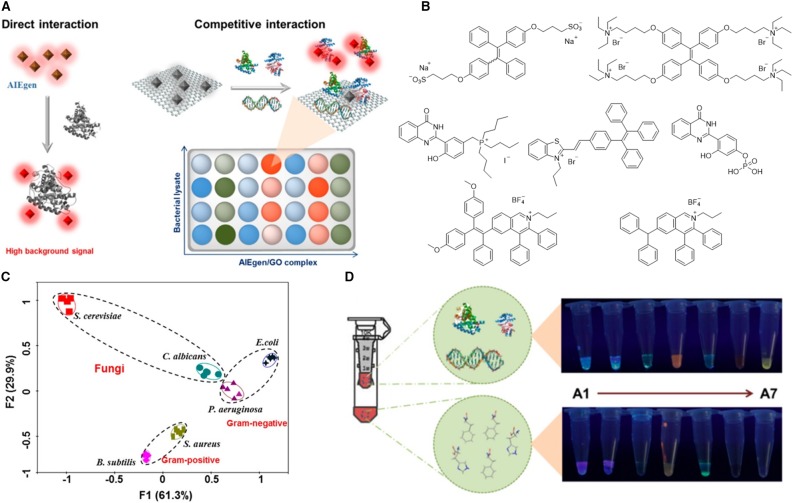
**(A)** Schematic illustration of the two strategies for constructing sensor arrays. The left represents the direct interaction between AIEgens and biomolecules, and the right demonstrates the competitive interaction among AIEgen, biomolecules, and GO. **(B)** Molecular structures of the selected AIEgens. **(C)** Principal component analysis results of the formed patterns generated from six microbial lysates. F1 and F2 are the top two rates of contribution in PCA analysis. **(D)** Separation of biomolecules into a large molecule portion (molecular weight >3,000) and a small molecule portion (molecular weight <3,000) with a ultrafiltration membrane. The fluorescence images of these two portions after incubation with AIEgens. Reproduced from Shen et al. (2018) with permission. Copyright 2018 American Chemical Society.

Besides alive microbes, their lysates are also employed for detection of bacteria. Microbial lysate detection is advantageous due to its stability during storage, less threat to the operator, insight into the microbial components, which are important for strain identification and development of microbial therapy. Shen et al. (2018) developed a fluorescent array for microbial lysates identification by employing AIEgens as the fluorescent sensors. Direct mixing of AIEgens with lysates usually results in high background emission, because some non-selective binding can also turn on the emission of some AIEgens. Thus, Shen et al. (2018) employed a graphene oxide (GO)–involved sensing strategy (Figure 10A). GO is known to be a fluorescence quencher and exhibits strong affinity to a large quantity of biomolecules. GO can compete with AIEgens for analytes and thus increase the discrimination of the detection by avoiding low-affinity binding of AIEgens with analytes. To construct a fluorescence sensor array, 7 of 13 AIEgens are screened, which demonstrate high fluorescence recovery efficiency upon interaction with GO and microbial lysates. The structures of these selected AIEgens are listed in Figure 10B, including five positively charged AIEgens, one negatively charged AIEgen, and one neutral AIEgen. To demonstrate the feasibility of the sensor array, six microbes are employed, including two fungi (*Candida albicans* and *Saccharomyces cerevisiae*), two Gram-positive bacteria (*Staphylococcus aureus* and *Bacillus subtilis*), and two Gram-negative bacteria (*P. aeruginosa* and *E. coli*). After incubating the microbe lysates with AIEgens for 2 h, the fluorescence intensity of the samples is collected and subjected to PCA to generate a canonical plot (Figure 10C), from which six microbe clusters are well separated from each other. Interestingly, fungi situate at the upper-left; Gram-negative bacteria locate on the upper-right, and Gram-positive bacteria lie at the bottom areas in the figure. The authors further explore the role of different lysate components in the detection. By filtrating the lysate with ultrafiltration membrane, bacterial lysates are separated into two parts with molecular weights higher or lower than 3,000. Large biomolecules in the lysates exhibit strong fluorescence recovering than small biomolecules, suggestive of the dominant role of large biomolecules, such as proteins and DNAs, in the detection process (Figure 10D).

## Summary and Perspectives

In this review, several examples are chosen to illustrate the recent progress of employing AIEgens for building up sensor arrays for identification of biological subjects, including biomolecules and bacteria. The results clearly demonstrate the excellent identification accuracy, low environmental interferences, and high identification efficiency of this approach. We believe array-based sensing employing AIEgens as sensors will become a future trend for analyte identification and will benefit our daily life. To achieve this goal, future research efforts can be devoted to the following areas: (1) mechanistic insight: fluorescent arrays produce array of signal, which are closely related to the interactions of sensors with analytes. Current investigations focus on the accuracy of identification but ignore these interactions. Statistical methods that can extract information on how the fluorescent pattern is produced are beneficial for the mechanistic understanding of probe–analyte interaction and contribute to the future development of biological probes. (2) Multiple-channel signal: the combination of multiple-channel signal will reduce the size of sensor array. As in Zhang and Li's system, by detecting fluorescence intensity and photonic property, one single sphere is capable of sensing multiple analytes. Research efforts on such multiple signals may be a further trend of research. (3) Clinical diagnosis: array-based sensing is featured with its workability in a complex environment, which enables their potential in clinical diagnosis. More clinical tests will be developed to extend the application scope of this method.

## Author Contributions

EZ and PL prepared the Figures for the work. EZ and SC wrote the draft of the review. YX and GZ revised the draft. All the authors discussed and decided on the topic of the review.

## Conflict of Interest

The authors declare that the research was conducted in the absence of any commercial or financial relationships that could be construed as a potential conflict of interest.
